# Analysis of Linear Antibody Epitopes on Factor H and CFHR1 Using Sera of Patients with Autoimmune Atypical Hemolytic Uremic Syndrome

**DOI:** 10.3389/fimmu.2017.00302

**Published:** 2017-03-30

**Authors:** Eszter Trojnár, Mihály Józsi, Katalin Uray, Dorottya Csuka, Ágnes Szilágyi, Danko Milosevic, Vesna D. Stojanović, Brankica Spasojević, Krisztina Rusai, Thomas Müller, Klaus Arbeiter, Kata Kelen, Attila J. Szabó, György S. Reusz, Satu Hyvärinen, T. Sakari Jokiranta, Zoltán Prohászka

**Affiliations:** ^1^3rd Department of Internal Medicine, Research Laboratory, Semmelweis University, Budapest, Hungary; ^2^MTA-ELTE “Lendület” Complement Research Group, Department of Immunology, Eötvös Loránd University, Budapest, Hungary; ^3^MTA-ELTE Research Group of Peptide Chemistry, Hungarian Academy of Sciences, Eötvös Loránd University, Budapest, Hungary; ^4^Department of Pediatric Nephrology, Dialysis and Transplantation, University of Zagreb, School of Medicine, University Hospital Center Zagreb, Zagreb, Croatia; ^5^Medical Faculty, Institute for Child and Youth Health Care of Vojvodina, University of Novi Sad, Novi Sad, Serbia; ^6^University Children’s Hospital, Nephrology, Dialysis and Transplantation Unit, Belgrade, Serbia; ^7^Department of Pediatrics, Medical University of Vienna, Vienna, Austria; ^8^1st Department of Pediatrics, Semmelweis University, Budapest, Hungary; ^9^Research Programs Unit, Immunobiology, University of Helsinki, Helsinki University Central Hospital, Helsinki, Finland

**Keywords:** atypical hemolytic uremic syndrome, factor H autoantibody, epitope mapping, CFHR1, complement factor H

## Abstract

**Introduction:**

In autoimmune atypical hemolytic uremic syndrome (aHUS), the complement regulator factor H (FH) is blocked by FH autoantibodies, while 90% of the patients carry a homozygous deletion of its homolog complement FH-related protein 1 (CFHR1). The functional consequence of FH-blockade is widely established; however, the molecular basis of autoantibody binding and the role of CFHR1 deficiency in disease pathogenesis are still unknown. We performed epitope mapping of FH to provide structural insight in the autoantibody recruitment on FH and potentially CFHR1.

**Methods:**

Eight anti-FH positive aHUS patients were enrolled in this study. With overlapping synthetic FH and CFHR1 peptides, we located the amino acids (aa) involved in binding of acute and convalescence stage autoantibodies. We confirmed the location of the mapped epitopes using recombinant FH domains 19–20 that carried single-aa substitutions at the suspected antibody binding sites in three of our patients. Location of the linear epitopes and the introduced point mutations was visualized using crystal structures of the corresponding domains of FH and CFHR1.

**Results:**

We identified three linear epitopes on FH (aa1157–1171; aa1177–1191; and aa1207–1226) and one on CFHR1 (aa276–290) that are recognized both in the acute and convalescence stages of aHUS. We observed a similar extent of autoantibody binding to the aHUS-specific epitope aa1177–1191 on FH and aa276–290 on CFHR1, despite seven of our patients being deficient for CFHR1. Epitope mapping with the domain constructs validated the location of the linear epitopes on FH with a distinct autoantibody binding motif within aa1183–1198 in line with published observations.

**Summary:**

According to the results, the linear epitopes we identified are located close to each other on the crystal structure of FH domains 19–20. This tertiary configuration contains the amino acids reported to be involved in C3b and sialic acid binding on the regulator, which may explain the functional deficiency of FH in the presence of autoantibodies. The data we provide identify the exact structures involved in autoantibody recruitment on FH and confirm the presence of an autoantibody binding epitope on CFHR1.

## Introduction

Atypical hemolytic uremic syndrome (aHUS) is a rare but life-threatening disease. It is characterized by the dysregulation of the complement alternative pathway due to mutations of the genes encoding complement factors and regulators, or autoantibodies directed against the regulator factor H (FH) (1, 2). Autoimmune aHUS usually evolves in children and adolescents and accounts for approximately 10% of all aHUS cases in the Western world (3–5). However, in an Indian cohort an incidence rate of 56% was reported, but the reason for this high frequency of autoantibody positivity remains to be explained (6). Autoimmune aHUS has a high relapse rate and risk of developing end stage renal disease (7–10).

Factor H consists of 20 homologous domains termed short consensus repeats (SCRs). It acts as a regulator of the complement alternative pathway (11–13) by exerting its cofactor and decay-accelerating activities through the N terminal SCR domains 1–4 and host discrimination (*via* sialic acid/glycosaminoglycan and C3b/C3d binding) through the C-terminal domains 19–20 (14, 15). The key characteristics of autoimmune aHUS are the FH autoantibodies that block FH (16), upon which the regulator is unable to restrain complement activation on host tissues. The autoimmune form of aHUS is linked to the deficiency of complement factor H-related (CFHR) proteins 1 and 3 (17) that have a yet unexplained role in the pathogenesis. While the frequency of heterozygous deletion of CFHR1 is similar in healthy individuals and aHUS patients, homozygous deletion of this protein is strongly associated with aHUS and was described in 82–88% of patients with FH autoantibodies (4, 6, 18–20). CFHR1 has a similar domain structure to that of FH, and SCR domain 5 of CFHR1 differs from FH SCR domain 20 in only two amino acids, whereas CFHR1 domain 4 and FH domain 19 are exactly the same (21). FH carries a serine at amino acid position 1191 and a valine at position 1197, while CFHR1 contains a leucine and an alanine at the corresponding positions (residue 290 and 296). Together with other CFHRs and FH-like protein 1, they form the FH protein family (22). Although CFHR1 competes with FH in C3b binding (23) and may neutralize the FH autoantibodies *in vitro* (24), it is currently unknown whether CFHR1 has a causative role in antibody production and how its deletion may contribute to the manifestation of aHUS.

Whereas the functional consequence of the antibody binding to FH is widely studied, little is known about the fine epitope specificity of the autoantibodies. Based on recent observations (25, 26), we hypothesized that the major antibody binding site is located on SCR domains 19–20 of FH, although other domains may also be recognized by aHUS-associated FH autoantibodies (19, 25, 27). Despite recent progress in the structural exploration of antibody binding to the folded FH domains (27, 28), we lack detailed knowledge of where the aHUS-associated FH epitopes are localized at the amino acid level. To answer this question, we performed fine epitope mapping using point-mutated FH domains and linear epitope mapping with overlapping synthetic peptides.

We further compared the epitopes of the autoantibodies on FH versus CFHR1. Our hypothesis was that aHUS-specific linear epitopes are also present on CFHR1, based on its homology to FH and its described cross-reactivity with the FH autoantibodies (24).

## Materials and Methods

### Patients and Serum Samples

For linear epitope mapping experiments leftover sera of children with treatment-naive, acute autoimmune aHUS were used. Inclusion criteria were as follows: presence of HUS [evidence of microangiopathic hemolytic anemia, evidence of renal injury, and evidence of thrombocytopenia (<150 G/L)] with an anti-FH autoantibody level >110 AU/ml (29). Exclusion criteria: HUS in convalescence (lack of hemolysis and lack of thrombocytopenia) and/or ongoing active treatment of HUS (either of the following: plasmapheresis, corticosteroids, cyclophosphamide, and rituximab) and/or lack of available serum sample (Figure S1 in Supplementary Material). Finally, eight patients could be enrolled in our investigations of which seven of eight had convalescence phase sample available (Table 1). Convalescence phase serum collection was done 6–12 months after the termination of any specific treatment of the patients. Samples of control children (median age of 9 years) were collected from leftover serum specimens from patients, who were admitted to the 1st Department of Pediatrics at Semmelweis University upon distinct indications, and detailed laboratory analysis (including inflammatory markers) did not reveal pathological findings. All control children were negative (below cutoff) for anti-FH. Patient enrollment was closed in September 2016. This study was carried out in accordance with the Helsinki Declaration, the study was approved by the Ethics Committee on Human Research in Budapest (8361-1/2011-EKU), and written informed consent was obtained from each subject.

**Table 1:** **Patients with the diagnosis of atypical HUS enrolled in this study who had positive ELISA results for anti-factor H (FH) antibodies (>110 AU/ml) at the time of presentation**.

Patient code	Age at disease onset (years)	Gender	FH autoantibody level in the acute phase of HUS (AU/ml)	FH autoantibody level in the convalescence phase of HUS (AU/ml)	MLPA analysis of CFHR1 and CFHR3
P1	6.5	Male	10,067	136	Homozygous deletion of CFHR1 and CFHR3
P2	8.5	Male	2,190	125	Homozygous deletion of CFHR1 and CFHR3
P3	10.5	Female	1,306	93	Homozygous deletion of CFHR1 and CFHR3
P4	8	Female	2,221	99	Homozygous deletion of CFHR1 and heterozygous deletion of CFHR3
P5	8	Male	2,725	213	Heterozygous deletion of CFHR1 and heterozygous deletion of CFHR3
P6	8	Male	209	55	Homozygous deletion of CFHR1 and CFHR3
P7	11	Male	329	89	Homozygous deletion of CFHR1 and CFHR3
P8	11	Female	9,152	No sample available	Homozygous deletion of CFHR1 and CFHR3

*Major clinical characteristics and results of multiplex ligation dependent-probe amplification (MLPA) are listed*.

### Determination of the FH Autoantibody Level in Patient Sera

Serum anti-FH IgG level was determined by an ELISA method described previously (1), with some modifications applied as follows. Nunc MaxiSorp plates (Nunc, Roskilde, Denmark) were coated with 1 μg/ml purified human FH (Calbiochem, San Diego, CA, USA). Serum samples were added at a dilution of 1:200 following blocking with PBS-1% BSA. As secondary antibody a rabbit anti-human IgG-HRP was used (Dako, Glostrup, Denmark) followed by the detection of bound IgG using 3,3′,5,5′-tetramethylbenzidine (TMB) with optical density (OD) read at λ = 450 nm (reference at λ = 620 nm). The assay was calibrated to a sample obtained as a kind gift from Dr. Dragon-Durey, and the cutoff value (>110 AU/ml) was determined according to the mean + 2 SD of 80 healthy individuals.

### Peptide Synthesis for Epitope Mapping

Fifteen amino acid-long peptides of FH SCR domains 19–20 and the CFHR1 region homolog to that of 1177–1211 on FH (amino acids 276–310 of CFHR1) were synthesized in duplicates on Mimotopes NCP gears (Clayton, VIC, Australia) according to Geysen’s method (30) as previously described (31). As control of peptide synthesis, three peptides were subjected to amino acid analysis, and the correct amino acid composition was verified. The amino acid sequence of each syntehtic peptide, as well as further details of the peptide synthesis are listed in Table S1 and Data Sheet S1 in Supplementary Material.

### Antibody Binding to the Immobilized Synthetic Peptides of FH and CFHR1

Serum antibody binding to overlapping synthetic peptides was determined by a modified ELISA as described previously (32). As a negative control peptide the heat shock protein (HSP) 60 fragment 480–489 was applied, based on our earlier observations, since this peptide showed the lowest binding with human sera in our past experiments (32). In the current experiments, we optimized the serum dilution to 1:1,000 and used a TMB detection system with OD read at λ = 450 nm (reference at λ = 620 nm). Data were normalized by the following formula: OD_sample_/OD_min_, where OD_sample_ is the mean of duplicate OD values of the test samples and OD_min_ represents the mean binding to the negative control HSP480–489 peptide. The interpretation of such a ratio can be done as fold changes over background. Epitope specific autoantibody binding in the acute phase and in convalescence serum samples is presented as OD_sample_/OD_min_ values, too.

### Antibody Binding to Recombinant FH 19–20 Mutants

Determination of serum antibody binding to the recombinant FH domains 19–20 displaying various single amino acid changes was performed with an ELISA-based method as previously described (25, 26).

### Location of the Epitopes and Point Mutations on the Folded FH and CFHR1 Structures

We analyzed the localization of the epitopes on the crystal structures of FH domains 19–20 [DOI:10.2210/pdb2g7i/pdb (33)] using the SWISS-PDB Viewer software [(34), http://www.expasy.org/spdbv/].

### Statistical Analysis

Data analysis was performed in the GraphPad Prism version 6.00 for Windows (GraphPad Software, La Jolla, CA, USA, www.graphpad.com). Autoantibody binding to the mutant FH domain constructs was compared to the wild type FH domains 19–20 with one-sample Wilcoxon signed-rank test. Anti-FH autoantibody binding in the independent samples was tested with Mann–Whitney test, whereas Wilcoxon matched-pairs signed-rank test was used to compare the acute and convalescence phase-autoantibody binding.

## Results

### Localization of Linear Autoantibody Epitopes on FH

**Figure 1:**
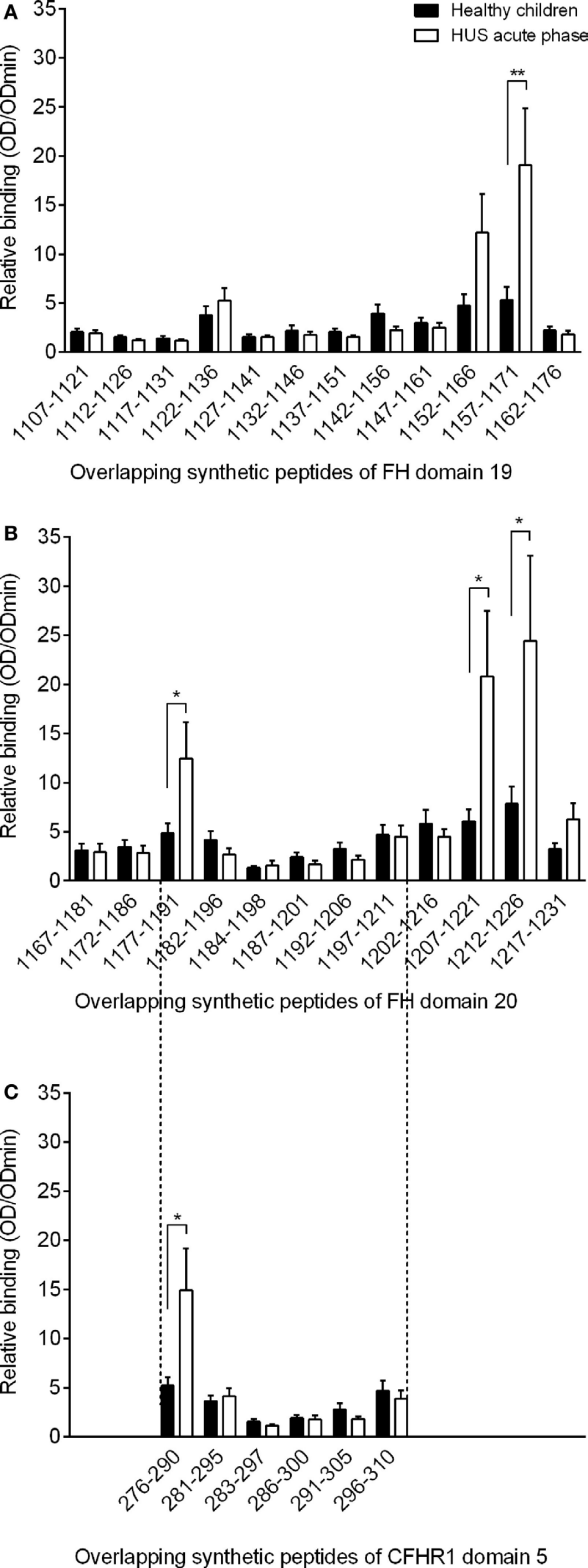
**Relative binding of serum factor H (FH) autoantibodies to the synthetic FH peptides in control patients and in acute phase of atypical hemolytic uremic syndrome**. The mapped FH domains were short consensus repeat (SCR) 19 **(A)**, SCR 20 **(B)** and the fragment of CFHR1 SCR domain 5 homolog to that of FH amino acids 1177–1211 **(C)**. We analyzed the sera of 10 control children (black bars) and 8 children in the acute phase of HUS (white bars), data represent mean of relative autoantibody binding of each group with SEM. Relative autoantibody binding is expressed as the ratio of OD_sample_/OD_min_, where OD_sample_ is the mean of duplicate optical density (OD) values of the test samples and OD_min_ is the mean binding to control HSP480–489 peptide that showed the lowest binding in our experiments. Numbering on the *x* axis represents the initial and final amino acid of each tested peptide. Difference in autoantibody binding to the indicated peptides was determined with Mann–Whitney test. Statistical significance is indicated by asterisks (*p < 0.05; **p < 0.01).

The synthetic peptides (Table S1 in Supplementary Material) were used for ELISA studies to determine autoantibody binding. Reactivity of serum autoantibodies to the synthetic peptides of 10 control children and 8 acute aHUS patients is shown in Figure 1. All serum samples of the eight aHUS patients were taken before the initiation of plasmapheresis; however, two of our patients received fresh frozen plasma prior to sampling. Peptides with a significantly increased autoantibody binding (patients versus control, Mann–Whitney test) contained either amino acids 1157–1171, located within FH domain 19, or amino acids 1177–1191 or 1207–1226, located within FH domain 20. The highest average binding was found with peptides containing residues 1212–1226. Even though average autoantibody binding to the linear peptides is shown, it is noteworthy that all three of the linear epitopes displayed increased autoantibody binding in the acute phase sera of every child.

### Epitope Specific Autoantibody Binding in Convalescence versus the Acute Phase of aHUS

**Figure 2:**
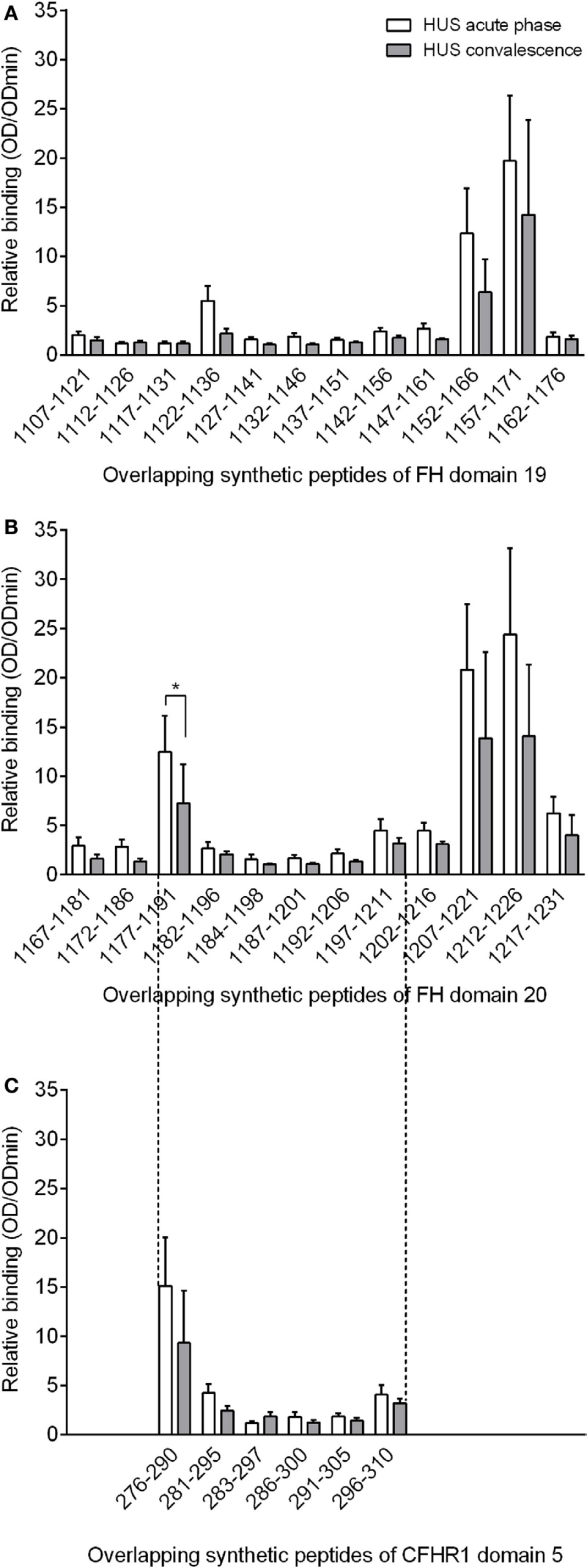
**Epitope specific relative anti-factor H autoantibody binding to the linear epitopes in convalescence (gray bars) versus the acute phase (white bars) of atypical hemolytic uremic syndrome**. The mapped FH domains were short consensus repeat (SCR) 19 **(A)**, SCR 20 **(B)** and the fragment of CFHR1 SCR domain 5 homolog to that of FH amino acids 1177-1211 **(C)**. Analysis of seven patients is shown as mean and SEM of the relative autoantibody binding (OD_sample_/OD_min_) of each group, where OD_sample_ is the mean of duplicate optical density (OD) values of the test samples and OD_min_ is the mean binding to control HSP480–489 peptide. Numbering on the *x* axis shows the initial and final amino acid of each tested peptide. Statistical analysis for the difference in autoantibody binding to the indicated peptides was performed using Wilcoxon matched-pairs signed-rank test (*p < 0.05).

We had the opportunity to test convalescence phase sera from seven of eight patients who were followed-up on a monthly basis after termination of therapy. Therapy of the children included plasmapheresis, immunosuppression with corticosteroids or cyclophosphamide, and/or rituximab. The convalescence phase samples were collected after a minimum follow-up period of 6 months in convalescence, during which the patients received no immunomodulation or any forms of plasma therapy. Albeit no relapse occurred in any of the enrolled patients, the level of free anti-FH IgG remained low-titer positive in three out of seven cases (Table 1). The epitope recognition pattern of the autoantibodies remained similar in the convalescence phase compared to what we had observed at the acute disease onset although the signals were weaker (Figure 2). The observed decline reached statistical significance by one of the peptides (peptide 1177–1191) although in case of all peptides a decrease of at least 25% was observed at the level of group mean.

### Comparison of FH Autoantibody Binding to Linear Epitopes on CFHR1 and FH

Since seven of our patients carried a homozygous and one a heterozygous deletion of CFHR1, we synthesized overlapping peptides of the region containing the two residues which are different in FH and CFHR1 (Table S1 in Supplementary Material). Supporting our observations with FH, we identified significant autoantibody binding to peptide 276–290 of CFHR1, which covered the exact same location as the previously identified autoantibody epitope on FH (peptide 1177–1191). Moreover, the serine–leucine exchange did not influence autoantibody binding since a very similar extent of binding was observed for the homologous peptides (Figures 1 and 2).

### Fine Epitope Mapping Using FH Domains 19–20 Displaying Point Mutations

**Figure 3:**
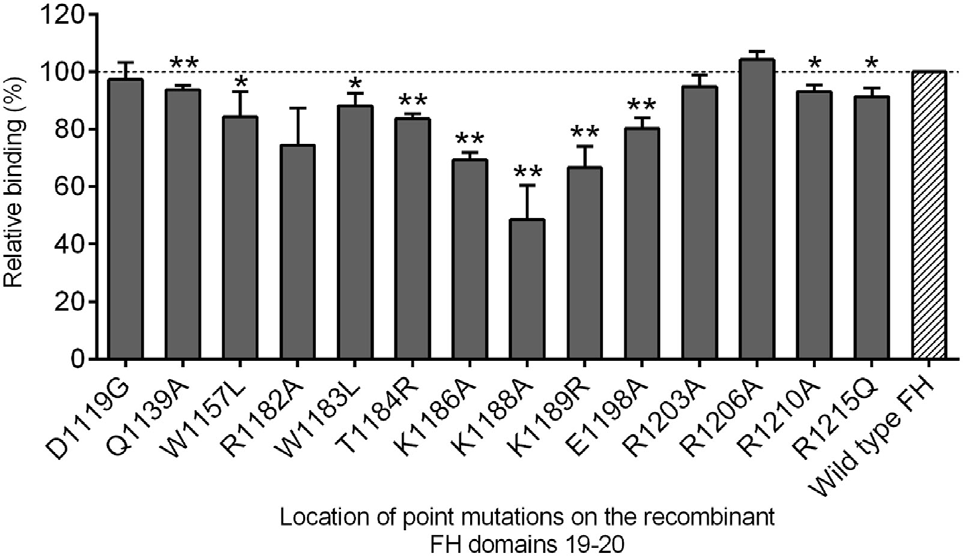
**Factor H (FH) autoantibody binding to single amino acid-substituted recombinant FH short consensus repeat (SCR) domains 19–20 compared to the wild type protein**. The amino acid substitutions are indicated on the *x* axis with capital letters, and numbers marking their location (gray bars) compared to the recombinant wild type FH SCRs 19–20 (striped bar). Binding is expressed in percent relative to that of the wild type (100%, intermittent line). We tested the sera of three patients in the acute phase of atypical hemolytic uremic syndrome; mean and SEM of three independent experiments are shown. Statistical analysis was performed with the one-sample Wilcoxon signed-rank test; statistical significance is indicated by asterisks (*p < 0.05; **p < 0.01).

We applied various recombinant FH19–20 constructs displaying single amino acid changes associated with aHUS to validate the results of the linear epitope mapping on the folded FH domains. To locate potential regions on FH19–20 where amino acid changes affect FH autoantibody binding (as compared to recombinant wild type FH), 14 different constructs were tested in ELISA (Figure 3) using serum samples of three patients. We observed the highest decrease in anti-FH binding in case of FH 19–20 construct displaying mutation at amino acid position 1188. A distinct antibody binding epitope appeared between amino acids (aa)1183–1198 with a symmetric gradual decline in antibody binding toward aa1188. Additional positions where anti-FH binding was decreased were detected on domain 19 (aa1139 and aa1157) and the C-terminal end of domain 20 (aa1210 and aa1215) concordant to the location of the identified linear epitopes.

### Location of the Identified Linear FH Epitopes on Crystal Structure of FH

**Figure 4:**
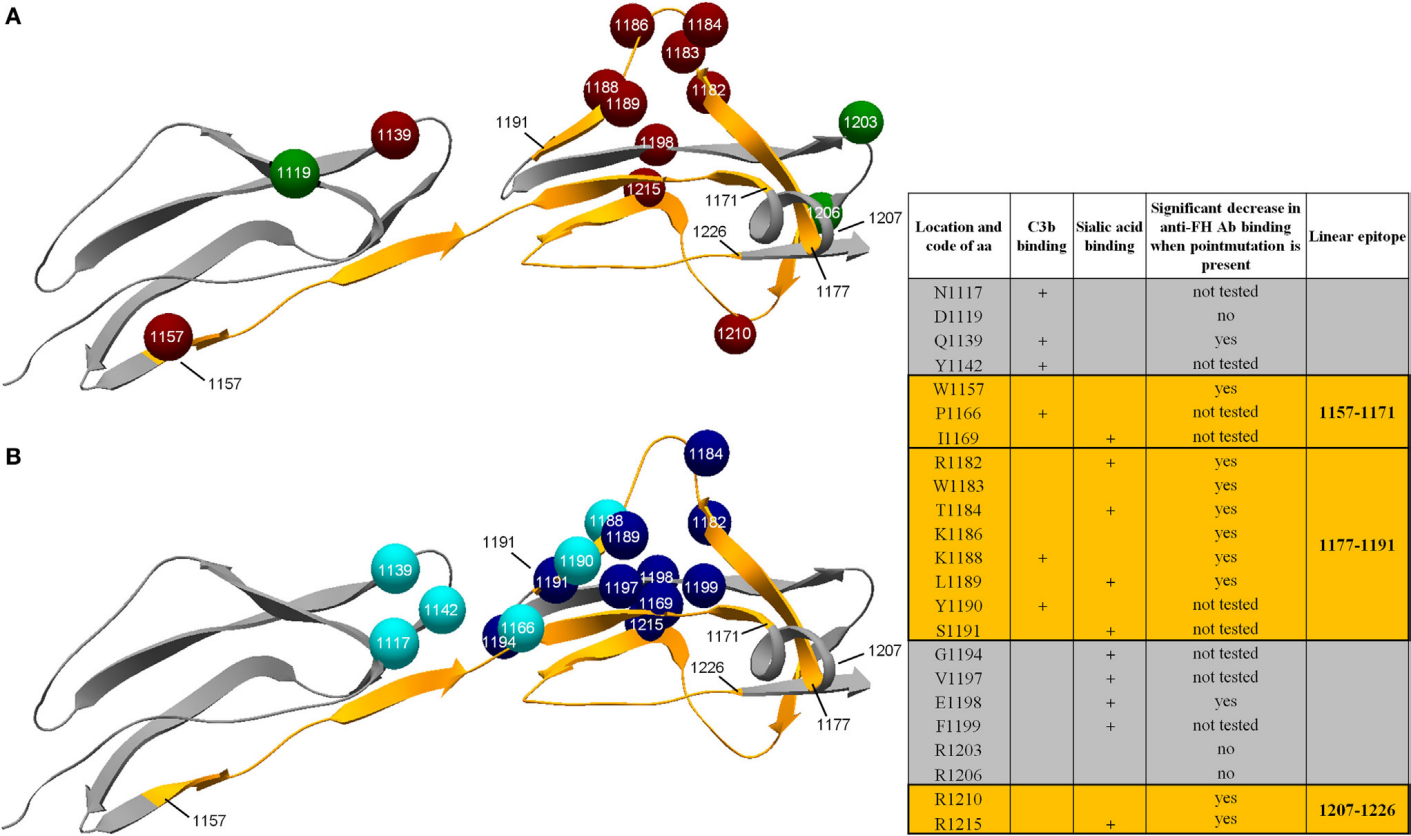
**Folded structure of complement factor H (FH) short consensus repeat domains 19–20**. Panels represent the ribbon model of the molecule obtained from the Protein Data Bank (pdb*2g7i*). Linear epitopes of the FH autoantibodies are highlighted with orange [amino acid (aa)1157–1171, aa1177–1191, and aa1207–1226], where the initial and final aa of each segment is indicated with black numbers. Arrowheads point toward the C-terminal end of the proteins. **(A)** The location of the generated point mutations is displayed as colorful spheres on the backbone of the protein, with white numbers indicating their location and colors their effect on autoantibody binding by FH (red: significantly decreased binding when the aa substitutions is present; green: no significant effect on binding). **(B)** Colorful spheres refer to aa forming the C3b (14, 35) (light blue) and sialic acid (15) (dark blue) binding sites of the molecule. Numbers within the spheres show the location of each aa.

We visualized the location of the linear epitopes in the tertiary structure of FH using its structure obtained from the Protein Data Bank (Figure 4). We also positioned the generated point mutations on the folded domains of the protein (Figure 4A). In the steric conformation, epitope 1157–1171 appears as a linear segment in the hinge region between FH domains 19 and 20, while epitopes 1177–1191 and especially 1207–1226 are in close sterical proximity with the C-terminal end of peptide 1157–1171. The homologous peptide on CFHR1 (276–290) had a similar structure to that of FH aa1177–1191 (data not shown).

## Discussion

In this study, we identified for the first time three linear, extended autoantibody binding epitopes on FH and one on CFHR1. These epitopes were recognized by both acute phase and convalescence phase serum autoantibodies of aHUS patients. We propose that the autoantibody binding site formed by the linear epitopes in the tertiary structure overlaps not only with the reported clustering of aHUS-associated FH mutations (Figure 4A) but also with the previously described location of FH fractions necessary for sialic acid and C3b binding (Figure 4B).

The epitope identified on CFHR1 confirms the previously observed cross-reactivity toward FH (18, 24), which may underline the role of CFHR1 in aHUS pathogenesis.

Results of the mutant domain and linear epitope mapping were concurrent on both domains 19 and 20 of FH. In line with published observations (25), we detected the biggest reduction in domain recognition when we introduced mutations to amino acids in the 1183–1198 region (Figure 3). This distinct binding motif overlaps the location of linear epitope 1177–1191, while on the C-terminal end of domain 20 the linear epitope 1207–1226 corroborates the reduced domain recognition of R1210A and R1215Q (Figure 4A).

The location of the identified epitopes corresponds to the known impairment of FH function in autoimmune aHUS. On one hand, the mutations Q1139A and two of the linear epitopes (1157–1171; 1177–1191) affect amino acids involved in C3b binding (14, 35), which matches the observed reduced C3b recognition of FH in the presence of autoantibodies (16). On the other hand, the epitope on the C-terminal end of domain 20 covers the reported heparin and sialic acid binding sites of the molecule (14, 15). This suggests a dwindled access of host surface sialic acids to autoantibody-bound FH, which could lead to subsequent loss of host recognition by the regulator. The simultaneous interference with both C3b binding and self-discrimination creates a basis for complement over-activation on host surfaces, most probably due to the inhibition of the C3b–sialic acid–FH complex formation as concluded by others (36). FH and related proteins are known to interact with the soluble pattern recognition molecules, pentraxins. The pentraxin-3 binding site on SCR20 of FH was recently located to involve amino acids 1180–1186 and 1198–1204 (37), which partially overlaps the identified autoantibody specific epitope 1177–1191. Due to inhibition of pentraxin binding, autoantibody-blocked FH may be unable to exert its complement regulatory activity at local sites of activation.

To outline the binding preference of the autoantibodies, we chose to include synthetic peptides of CFHR1 in the linear epitope mapping. Surprisingly, we detected a similar autoantibody recognition pattern on CFHR1 to that of FH in both controls and aHUS patients. The FH autoantibody specific epitope on CFHR1 (276–290) showed the same extent of autoantibody binding as its homolog on FH, although seven of our patients tested deficient for CFHR1. This observation is in line with the described cross-reactivity and neutralizing effect on CFHR1 of FH autoantibodies (18, 24), even though the C-terminal domains of CFHR1 are described to have lower avidity to the autoantibodies than those of FH (26). The concept that an induced neoepitope on FH similar in structure to CFHR1 drives autoantibody production (26) could explain the equivalent autoantibody binding, even though CFHR1 contains a leucine at position 290 instead of the serine present on FH at position 1191. However, as a limitation of our study, the synthetic peptides represented linear autoantibody binding sites with equal accessibility, while under physiological circumstances some of these epitopes may be hidden, especially in the cryptic conformation of FH.

There was a notable difference in the extent of autoantibody binding to the linear epitopes in the individual samples, which matched the FH autoantibody levels of the patients. However, all three of the identified linear epitopes showed increased autoantibody binding compared to the background reactivity in every patient’s serum. Although the level of epitope specific antibodies decreased in convalescence, the binding pattern remained the same with autoantibody positivity (>110 AU/ml) in three of our patients, but no relapse during the follow-up period. This finding, together with the observation that anti-FH autoantibodies might be present in healthy individuals (6) may further support the concept of a necessary environmental trigger event preceding the manifestation of the disease, not only by the first acute episode but also before relapses.

The identified epitopes are located close to each other on the folded domains of FH. The middle of fragment 1177–1191 protrudes from the surface of the protein (1185–1187 residues are highly accessible), then a few of its C-terminal residues form a short parallel structure with the 1165–1168 residues of the linear structure 1157–1171. The C-terminal epitope 1207–1226 forms a loop, centering in a turn structure of 1219–1222, and the residues of the turn are again close to the 1163–1167 residues of peptide 1157–1171: the C-terminal residues of 1177–1191 and the turn region of 1207–1226 sandwich the middle of the 1157–1171 sequence. This configuration of the linear epitopes overlaps the reported ternary complex of FH, sialic acids and the C3b thioester-containing domain (Figure 4B). The recognition of all three of the linear epitopes by the autoantibodies of every patient lets us suspect that wherever the autoantibody binding occurs, it either alters the conformation of this binding site or interferes with C3b and sialic acid recognition by FH through steric hindrance. This hypothesis is also substantiated by the clustering of aHUS-associated FH mutations at this region (26).

Taken together, we have shown that the aHUS-specific autoantibodies recognize three distinct linear epitopes on FH and one on CFHR1. The linear binding sites on FH are located close to each other in the tertiary structure, which may suggest the formation of a conformational antibody binding site during *in vivo* folding. However, this assumption requires further experimental confirmation. The novelty of this work lies in the testing of autoantibody binding to factory H in a direct way, using synthetic peptides of the regulator and also CFHR1. The presence of the autoantibody binding epitopes was validated with the binding assays performed with recombinant FH domains 19–20 with point mutations. The lack of significant decrease in the epitope specific autoantibody binding to all, but one epitope in the convalescence phase of aHUS indicates the presence of additional factors that trigger relapses. The presence of an autoantibody specific epitope on CFHR1 underlines its role in aHUS pathogenesis, although its exact function is yet to be defined. Observations of this study contribute to the accurate mapping of the autoantibody binding site on FH and CFHR1, which in the long term may help us to design specific inhibitors thus preserving FH function and also to explore in detail the necessary factors involved in aHUS pathogenesis.

## Author Contributions

Study concept and design: ZP and MJ; experimental procedures: ET, KU, MJ, DC, ASz, SH, and TJ; acquisition of data: DM, VS, BS, KR, TM, KA, KK, ASz, GR, KU, SH, and TJ; analysis and interpretation of data: ET, ZP, KU, MJ, DC, ASz, DM, VS, BS, KR, TM, KA, KK, SzA, GR, SH, and TJ; critical writing of the manuscript: ET and ZP; critical revision of the manuscript for important intellectual content: all athors; study supervision: ZP and MJ; acquisition of funding: MJ, TJ, and ZP.

## Conflict of Interest Statement

The authors declare that the research was conducted in the absence of any commercial or financial relationships that could be construed as a potential conflict of interest.

## References

[1] Dragon-Durey MA Loirat C Cloarec S Macher MA Blouin J Nivet H Anti-Factor H autoantibodies associated with atypical hemolytic uremic syndrome. J Am Soc Nephrol (2005) 16(2):555–63. 10.1681/ASN.2004050380 15590760

[2] Kavanagh D Goodship TH Richards A. Atypical hemolytic uremic syndrome. Semin Nephrol (2013) 33(6):508–30. 10.1016/j.semnephrol.2013.08.003 24161037 PMC3863953

[3] Skerka C Jozsi M Zipfel PF Dragon-Durey MA Fremeaux-Bacchi V. Autoantibodies in haemolytic uraemic syndrome (HUS). Thromb Haemost (2009) 101(2):227–32. 10.1160/TH08-05-0322 19190803

[4] Hofer J Janecke AR Zimmerhackl LB Riedl M Rosales A Giner T Complement factor H-related protein 1 deficiency and factor H antibodies in pediatric patients with atypical hemolytic uremic syndrome. Clin J Am Soc Nephrol (2013) 8(3):407–15. 10.2215/CJN.01260212 23243267 PMC3586960

[5] Nester CM Barbour T de Cordoba SR Dragon-Durey MA Fremeaux-Bacchi V Goodship TH Atypical aHUS: state of the art. Mol Immunol (2015) 67(1):31–42. 10.1016/j.molimm.2015.03.246 25843230

[6] Sinha A Gulati A Saini S Blanc C Gupta A Gurjar BS Prompt plasma exchanges and immunosuppressive treatment improves the outcomes of anti-factor H autoantibody-associated hemolytic uremic syndrome in children. Kidney Int (2014) 85(5):1151–60. 10.1038/ki.2013.373 24088957

[7] Noris M Remuzzi G Atypical hemolytic-uremic syndrome. N Engl J Med (2009) 361(17):1676–87. 10.1056/NEJMra0902814 19846853

[8] Noris M Remuzzi G Glomerular diseases dependent on complement activation, including atypical hemolytic uremic syndrome, membranoproliferative glomerulonephritis, and C3 glomerulopathy: core curriculum 2015. Am J Kidney Dis (2015) 66(2):359–75. 10.1053/j.ajkd.2015.03.040 26032627 PMC4528072

[9] Dragon-Durey MA Blanc C Garnier A Hofer J Sethi SK Zimmerhackl LB. Anti-factor H autoantibody-associated hemolytic uremic syndrome: review of literature of the autoimmune form of HUS. Semin Thromb Hemost (2010) 36(6):633–40. 10.1055/s-0030-1262885 20865640

[10] Dragon-Durey MA Sethi SK Bagga A Blanc C Blouin J Ranchin B Clinical features of anti-factor H autoantibody-associated hemolytic uremic syndrome. J Am Soc Nephrol (2010) 21(12):2180–7. 10.1681/ASN.2010030315 21051740 PMC3014031

[11] Ferreira VP Pangburn MK Cortes C. Complement control protein factor H: the good, the bad, and the inadequate. Mol Immunol (2010) 47(13):2187–97. 10.1016/j.molimm.2010.05.007 20580090 PMC2921957

[12] Schmidt CQ Herbert AP Hocking HG Uhrin D Barlow PN. Translational mini-review series on complement factor H: structural and functional correlations for factor H. Clin Exp Immunol (2008) 151(1):14–24. 10.1111/j.1365-2249.2007.03553.x 18081691 PMC2276926

[13] de Cordoba SR de Jorge EG. Translational mini-review series on complement factor H: genetics and disease associations of human complement factor H. Clin Exp Immunol (2008) 151(1):1–13. 10.1111/j.1365-2249.2007.03552.x 18081690 PMC2276932

[14] Kajander T Lehtinen MJ Hyvarinen S Bhattacharjee A Leung E Isenman DE Dual interaction of factor H with C3d and glycosaminoglycans in host-nonhost discrimination by complement. Proc Natl Acad Sci U S A (2011) 108(7):2897–902. 10.1073/pnas.1017087108 21285368 PMC3041134

[15] Blaum BS Hannan JP Herbert AP Kavanagh D Uhrin D Stehle T. Structural basis for sialic acid-mediated self-recognition by complement factor H. Nat Chem Biol (2015) 11(1):77–82. 10.1038/nchembio.1696 25402769

[16] Jozsi M Strobel S Dahse HM Liu WS Hoyer PF Oppermann M Anti factor H autoantibodies block C-terminal recognition function of factor H in hemolytic uremic syndrome. Blood (2007) 110(5):1516–8. 10.1182/blood-2007-02-071472 17495132

[17] Zipfel PF Edey M Heinen S Jozsi M Richter H Misselwitz J Deletion of complement factor H-related genes CFHR1 and CFHR3 is associated with atypical hemolytic uremic syndrome. PLoS Genet (2007) 3(3):e41. 10.1371/journal.pgen.0030041 17367211 PMC1828695

[18] Moore I Strain L Pappworth I Kavanagh D Barlow PN Herbert AP Association of factor H autoantibodies with deletions of CFHR1, CFHR3, CFHR4, and with mutations in CFH, CFI, CD46, and C3 in patients with atypical hemolytic uremic syndrome. Blood (2010) 115(2):379–87. 10.1182/blood-2009-05-221549 19861685 PMC2829859

[19] Jozsi M Licht C Strobel S Zipfel SL Richter H Heinen S Factor H autoantibodies in atypical hemolytic uremic syndrome correlate with CFHR1/CFHR3 deficiency. Blood (2008) 111(3):1512–4. 10.1182/blood-2007-09-109876 18006700

[20] Dragon-Durey MA Blanc C Marliot F Loirat C Blouin J Sautes-Fridman C The high frequency of complement factor H related CFHR1 gene deletion is restricted to specific subgroups of patients with atypical haemolytic uraemic syndrome. J Med Genet (2009) 46(7):447–50. 10.1136/jmg.2008.064766 19435718

[21] Abarrategui-Garrido C Martinez-Barricarte R Lopez-Trascasa M de Cordoba SR Sanchez-Corral P. Characterization of complement factor H-related (CFHR) proteins in plasma reveals novel genetic variations of CFHR1 associated with atypical hemolytic uremic syndrome. Blood (2009) 114(19):4261–71. 10.1182/blood-2009-05-223834 19745068

[22] Medjeral-Thomas N Pickering MC. The complement factor H-related proteins. Immunol Rev (2016) 274(1):191–201. 10.1111/imr.12477 27782332

[23] Goicoechea de Jorge E Caesar JJ Malik TH Patel M Colledge M Johnson S Dimerization of complement factor H-related proteins modulates complement activation in vivo. Proc Natl Acad Sci U S A (2013) 110(12):4685–90. 10.1073/pnas.1219260110 23487775 PMC3606973

[24] Strobel S Abarrategui-Garrido C Fariza-Requejo E Seeberger H Sanchez-Corral P Jozsi M. Factor H-related protein 1 neutralizes anti-factor H autoantibodies in autoimmune hemolytic uremic syndrome. Kidney Int (2011) 80(4):397–404. 10.1038/ki.2011.152 21677636

[25] Nozal P Bernabeu-Herrero ME Uzonyi B Szilagyi A Hyvarinen S Prohaszka Z Heterogeneity but individual constancy of epitopes, isotypes and avidity of factor H autoantibodies in atypical hemolytic uremic syndrome. Mol Immunol (2016) 70:47–55. 10.1016/j.molimm.2015.12.005 26703217

[26] Bhattacharjee A Reuter S Trojnar E Kolodziejczyk R Seeberger H Hyvarinen S The major autoantibody epitope on factor H in atypical hemolytic uremic syndrome is structurally different from its homologous site in factor H-related protein 1, supporting a novel model for induction of autoimmunity in this disease. J Biol Chem (2015) 290(15):9500–10. 10.1074/jbc.M114.630871 25659429 PMC4392255

[27] Blanc C Roumenina LT Ashraf Y Hyvarinen S Sethi SK Ranchin B Overall neutralization of complement factor H by autoantibodies in the acute phase of the autoimmune form of atypical hemolytic uremic syndrome. J Immunol (2012) 189(7):3528–37. 10.4049/jimmunol.1200679 22922817

[28] Strobel S Hoyer PF Mache CJ Sulyok E Liu WS Richter H Functional analyses indicate a pathogenic role of factor H autoantibodies in atypical haemolytic uraemic syndrome. Nephrol Dial Transplant (2010) 25(1):136–44. 10.1093/ndt/gfp388 19666655

[29] Szilagyi A Kiss N Bereczki C Talosi G Racz K Turi S The role of complement in *Streptococcus pneumoniae*-associated haemolytic uraemic syndrome. Nephrol Dial Transplant (2013) 28(9):2237–45. 10.1093/ndt/gft198 23787556

[30] Geysen HM Meloen RH Barteling SJ. Use of peptide synthesis to probe viral antigens for epitopes to a resolution of a single amino acid. Proc Natl Acad Sci U S A (1984) 81(13):3998–4002. 10.1073/pnas.81.13.3998 6204335 PMC345355

[31] Uray K Hudecz F Fust G Prohaszka Z. Comparative analysis of linear antibody epitopes on human and mycobacterial 60-kDa heat shock proteins using samples of healthy blood donors. Int Immunol (2003) 15(10):1229–36. 10.1093/intimm/dxg122 13679392

[32] Fust G Uray K Bene L Hudecz F Karadi I Prohaszka Z. Comparison of epitope specificity of anti-heat shock protein 60/65 IgG type antibodies in the sera of healthy subjects, patients with coronary heart disease and inflammatory bowel disease. Cell Stress Chaperones (2012) 17(2):215–27. 10.1007/s12192-011-0301-7 22038196 PMC3273563

[33] Jokiranta TS Jaakola VP Lehtinen MJ Parepalo M Meri S Goldman A. Structure of complement factor H carboxyl-terminus reveals molecular basis of atypical haemolytic uremic syndrome. EMBO J (2006) 25(8):1784–94. 10.1038/sj.emboj.7601052 16601698 PMC1440827

[34] Guex N Peitsch MC. SWISS-MODEL and the Swiss-PdbViewer: an environment for comparative protein modeling. Electrophoresis (1997) 18(15):2714–23. 10.1002/elps.1150181505 9504803

[35] Morgan HP Schmidt CQ Guariento M Blaum BS Gillespie D Herbert AP Structural basis for engagement by complement factor H of C3b on a self surface. Nat Struct Mol Biol (2011) 18(4):463–70. 10.1038/nsmb.2018 21317894 PMC3512577

[36] Hyvarinen S Meri S Jokiranta TS. Disturbed sialic acid recognition on endothelial cells and platelets in complement attack causes atypical hemolytic uremic syndrome. Blood (2016) 127(22):2701–10. 10.1182/blood-2015-11-680009 27006390

[37] Kopp A Strobel S Tortajada A Rodriguez de Cordoba S Sanchez-Corral P Prohaszka Z Atypical hemolytic uremic syndrome-associated variants and autoantibodies impair binding of factor h and factor h-related protein 1 to pentraxin 3. J Immunol (2012) 189(4):1858–67. 10.4049/jimmunol.1200357 22786770

