# Supplementary material for: Analysis of Linear Antibody Epitopes on Factor H and CFHR1 Using Sera of Patients with Autoimmune Atypical Hemolytic Uremic Syndrome
Source: Front Immunol. 2017 Mar 30;8:302. doi: 10.3389/fimmu.2017.00302 (PMC5371605; doi:10.3389/fimmu.2017.00302)
Supplement: Supplementary file 1 [file Table_1.DOCX]

| **CFH domain 19** | **CFH domain 20** | **CFHR1** | **Negative control HSP60** |
| --- | --- | --- | --- |
| ^1107^GKCGPPPPIDNGDIT^1121^****** | ^1167^CVISREIMENYNIAL^1181^ |  | ^480^AKNAGVEGSL^489^ |
| ^1112^PPPIDNGDITSFPLS^1126^ | ^1172^EIMENYNIALRWTAK^1186^ |  |  |
| ^1117^NGDITSFPLSVYAPA^1131^ | ^1177^YNIALRWTAKQKLYS^1191^ | ^276^YNIALRWTAKQKLY**L**^290^ |  |
| ^1122^SFPLSVYAPASSVEY^1136^ | ^1182^RWTAKQKLYSRTGES^1196^ | ^281^RWTAKQKLY**L**RTGES^295^ |  |
| ^1127^VYAPASSVEYQCQNL^1141^****** | ^1184^TAKQKLYSRTGESVE^1198^ | ^283^TAKQKLY**L**RTGES**A**E^297^ |  |
| ^1132^SSVEYQCQNLYQLEG^1146^ | ^1187^QKLYSRTGESVEFVC^1201^ | ^286^QKLY**L**RTGES**A**EFVC^300^ |  |
| ^1137^QCQNLYQLEGNKRIT^1151^ | ^1192^RTGESVEFVCKRGYR^1206^ | ^291^RTGES**A**EFVCKRGYR^305^ |  |
| ^1142^YQLEGNKRITCRNGQ^1156^ | ^1197^VEFVCKRGYRLSSRS^1211^ | ^296^**A**EFVCKRGYRLSSRS^310^ |  |
| ^1147^NKRITCRNGQWSEPP^1161^****** | ^1202^KRGYRLSSRSHTLRT^1216^ |  |  |
| ^1152^CRNGQWSEPPKCLHP^1166^ | ^1207^LSSRSHTLRTTCWDG^1221^ |  |  |
| ^1157^WSEPPKCLHPCVISR^1171^ | ^1212^HTLRTTCWDGKLEYP^1226^ |  |  |
| ^1162^KCLHPCVISREIMEN^1176^ | ^1217^TCWDGKLEYPTCAKR^1231^ |  |  |

**Supplementary Table 1.** Amino acid sequence of the synthetic peptides. Numbers indicate the position of the initial and last amino acid of each synthetic peptide, capitals refer to the single letter amino acid codes. Bold letters indicate the differences between the homologous FH domain 20 and CFHR1 peptides. Peptide sequences labelled with ****** have been prepared in an additional copy and subjected to amino acid analysis, where the correct amino acid composition was verified.
