# Supplementary material for: Analysis of Linear Antibody Epitopes on Factor H and CFHR1 Using Sera of Patients with Autoimmune Atypical Hemolytic Uremic Syndrome
Source: Front Immunol. 2017 Mar 30;8:302. doi: 10.3389/fimmu.2017.00302 (PMC5371605; doi:10.3389/fimmu.2017.00302)
Supplement: Supplementary file 2 [file Data_Sheet_1.DOCX]

**Supplementary material:**

Peptide synthesis in detail

The N^α^-Fmoc protecting group was removed with 2 % piperidine / 2 % 1,8-diazabicyclo(5.4.0)undec-7-ene in DMF, and the protected amino acid residues were coupled with N,N’-diisopropylcarbodiimide/1-hydroxybenzotriazol. The side chain protecting groups were the following: aspartate, glutamate: tert-butyl ether; threonine, serine, tyrosine: tert-butyl ether; arginine: 2,2,4,6,7-pentamethyldihydrobenzofuran-5-sulfonyl (Pbf); histidine, asparagine, glutamine: triphenylmethyl; cysteine: acetamidomethyl. The protecting groups (with the exception of acetamidomethyl) were removed by a solution of trifluoroacetic acid–water–phenol–thioanisole–1,2-ethanedithiol 90:5:7.5:5:2.5 (V/V/m/V/V%), while the peptides remained covalently immobilized to the gears. The only remainder protecting group, acetamidomethyl on cysteine was masking the thiol-group of cysteine, which forms a disulfide bond in the folded protein. As control of peptide synthesis, three peptides were subjected to amino acid analysis, and the correct amino acid composition was verified. The amino acid sequence of each synthetic peptide is listed in Table S1 in Supplementary Material.
