# Supplementary figures and images for: Analysis of Linear Antibody Epitopes on Factor H and CFHR1 Using Sera of Patients with Autoimmune Atypical Hemolytic Uremic Syndrome
Source: Front Immunol. 2017 Mar 30;8:302. doi: 10.3389/fimmu.2017.00302 (PMC5371605; doi:10.3389/fimmu.2017.00302)

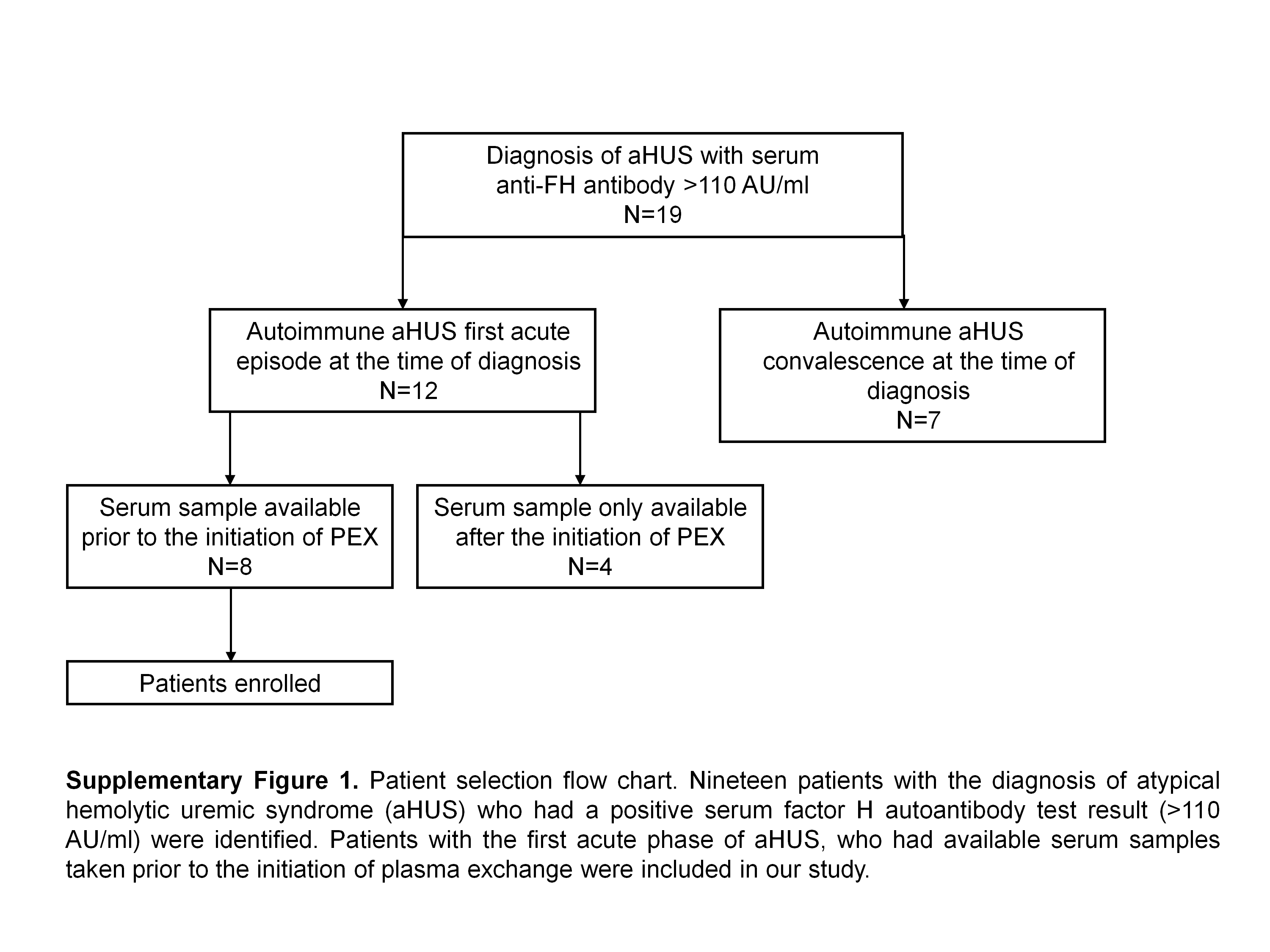

Supplement: Supplementary file 3 [file Image_1.TIF]
